# Manual of the Diseases of the Eye, for Students and General Practitioners

**Published:** 1901-09

**Authors:** 


Manual of the Diseases of the Eye, for students and general practitioners. With 243 original illustrations, including twelve colored figures. By Charles H. May, M. D., Chief of Clinic and Instructor in Ophthalmology, Eye Department, College of Physicians and Surgeons, New York. Pp. 406. Price, $1.25. New York: William Wood & Co. 1900.

The author well kept in mind the fact that he was writing a short practical manual for students and general practitioners. This book can be carried in the pocket, and yet it covers the fundamental facts in ophthalmology. The author is modest enough to not recommend this little volume as a substitute for larger works, but says in his preface it should be used "as a means of supplying a foundation to which further knowledge may be added by reference to more extensive and comprehensive text-books." T. J. B.

